# Protective effect of tanshinone IIA on the brain and its therapeutic time window in rat models of cerebral ischemia-reperfusion

**DOI:** 10.3892/etm.2014.1936

**Published:** 2014-08-27

**Authors:** QIQIANG TANG, RUODONG HAN, HAN XIAO, JUN LI, JILONG SHEN, QINGLI LUO

**Affiliations:** 1 Department of Neurology, Affiliated Provincial Hospital, Anhui Medical University, Hefei, Anhui 230022, P.R. China; 2 School of Pharmacy, Anhui Medical University, Hefei, Anhui 230032, P.R. China; 3 Institute of Clinical Pharmacology, Anhui Medical University, The Key Laboratories of Zoonoses and Pathogen Biology, Hefei, Anhui 230022, P.R. China

**Keywords:** tanshinone, middle cerebral artery occlusion, neuroprotection, time window

## Abstract

The aims of the present study were to investigate the protective effect of tanshinone IIA on the brain and its therapeutic time window in a rat model of cerebral ischemia-reperfusion. The rat model of cerebral ischemia-reperfusion was established by suture occlusion. In an initial experiment, male Sprague-Dawley (SD) rats were randomly divided into control cerebral ischemia-reperfusion rat model, tanshinone IIA1 (TSA1), tanshinone IIA4 (TSA4), tanshinone IIA6 (TSA6) and tanshinone IIA12 (TSA12) groups (n=8 per group). The rats in the control group were given 4 ml phosphate-buffered saline (PBS) intraperitoneally following suture occlusion. The other groups were respectively treated with 25 mg/kg tanshinone IIA intraperitoneally at 1, 4, 6 and 12 h following the initiation of reperfusion and once a day for a total of three days. The grades of neurologic impairment and volume of cerebral infarction of each group were measured 72 h after suture occlusion. In another experiment, 16 male SD rats were randomly divided into a 6 h reperfusion group and a 24 h reperfusion group following drug administration. The rats in each group were further divided into a control subgroup (4 ml PBS) and a tanshinone IIA subgroup (25 mg/kg). The rats were immediately administered their respective treatments following the establishment of the model. The rats were decapitated 6 and 24 h after the initiation of reperfusion. The expression levels of cytoplasmic thioredoxin (Trx-1) and mitochondrial thioredoxin (Trx-2) in the ischemic penumbra were determined by western blot analysis. The nitric oxide (NO) levels, and total NO synthase (tNOS) and inducible NO synthase (iNOS) activities in the rat blood were measured using a reagent kit. The changes in cerebral blood flow were evaluated by Doppler imaging. The grade of neurological impairment of the TSA1 group was statistically lower than that of the other groups (P<0.05). The cerebral infarction volume results showed that the volumes of infarction in the TSA1 and TSA4 groups were lower than those in the other groups (P<0.05). Tanshinone IIA significantly increased cerebral blood flow compared with that of the control group (P<0.05). Moreover, tanshinone IIA significantly increased the expression levels of Trx-1 and Trx-2 compared with those in the control group (P<0.05). Tanshinone IIA significantly decreased the NO levels and iNOS and tNOS activities compared with those of the control group (P<0.05). However, the iNOS activity in the rats in the 6 h reperfusion group was not statistically significantly different from that of the respective control group (P>0.05). Tanshinone IIA has a protective effect on the cranial nerves when administered during the initial stages of cerebral ischemia. This protective effect is associated with an improvement of cerebral blood flow as well as an increase in anti-oxygen radical and anti-inflammatory activities.

## Introduction

The morbidity of cerebral stroke, which is characterized by high paralysis and fatality rates, has markedly increased following improvements in living standards, lifestyle changes and population aging. These factors increase the financial burden on patients. Stroke is a leading cause of serious long-term disability in adults and is the second leading cause of death worldwide. The cerebral ischemia-reperfusion model is a recognized cerebral ischemic model. The main pathomechanism of ischemic cerebral injury includes excitable toxicity, oxidative stress, inflammation, cell apoptosis and mitochondrial dysfunction, which may lead to cell death within a few minutes (2,3). Cell death in the ischemic penumbra around the infarction is slower than that in the infarction area. Thus, saving the cells in the ischemic penumbra is an important target of studies concerning cerebral infarction treatment. Free radicals and oxygen radical scavengers that are closely associated with the physiology and pathological processes of the nerves, such as superoxides, hydroxide radicals, hydrogen peroxide, nitric oxide (NO), superoxide dismutase, thioredoxin (Trx) and Trx reductase, play important roles in cerebral injury (4,5). NO, which acts as an important messenger and effector molecule (6), is a type of endogenous medium for regulating neurotransmission, vasodilatation and nerve function. NO synthase (NOS) is a key enzyme for NO synthesis that exists in three types, namely, neuronal NOS (nNOS), endothelial NOS (eNOS) and inducible NOS (iNOS). nNOS and eNOS are activated by calcium ions and calmodulin, whereas iNOS is activated by inflammatory factors and endotoxins. The action time of iNOS is prolonged and iNOS catalyzes the synthesis of large amounts of NO (7). eNOS activity increases and catalyzes NO synthesis, thereby expanding brain blood vessels and increasing brain blood flow to protect the brain, at the very early stages of cerebral infarction (<2 h). The protective effect of eNOS disappears 2 h after cerebral infarction. nNOS catalyzes the synthesis of large quantities of NO that have a toxic effect on the brain during the early stage of cerebral infarction (2–4 h). iNOS may catalyze the synthesis of a large amount of NO, thus increasing glutamic acid toxicity and leading to tardive neuronal damage (8).

Trx is a type of redox-active macromolecular protein that has many important biological functions, including mediation of the transport of H^+^ ions in systems involving NADPH and Trx reductase. Trx plays a role in oxidoreduction due to its disulfide bond, acts as a biomarker of oxidative stress and participates in oxidative stress and cell apoptosis. Trx includes cytoplasmic Trx (Trx-1) and mitochondrial Trx (Trx-2) (9–12).

Tanshinone is a mixture of numerous compounds used for the treatment of cardiovascular and cerebrovascular diseases (13–15). Tanshinone IIA is the main active ingredient of tanshinone, which is a phenanthrenequinone derivative (16). Previous studies have confirmed the protective effect of tanshinone IIA on the brains of mice and rats undergoing cerebral ischemia-reperfusion (15,17). However, few studies exist on the effects of tanshinone IIA on early oxidative stress and on the timing of treatment following the occurrence of cardiovascular and cerebrovascular diseases. Thus, the present study was designed to elucidate the mechanism of the protective effect of tanshinone IIA on the brain and its optimum time of administration in a rat model of cerebral ischemia-reperfusion.

## Materials and methods

### Animals

#### Grouping I and drug administration

A total of 40 male Sprague-Dawley (SD) rats (clean grade, 200–220 g) provided by the Experimental Animal Centre of Anhui Medical University (Hefei, China) were randomly divided into five groups: control cerebral ischemia-reperfusion rat model, tanshinone IIA1 (TSA1), tanshinone IIA4 (TSA4), tanshinone IIA6 (TSA6) and tanshinone IIA12 (TSA12) groups. The rats in the control group were administered 4 ml phosphate-buffered saline (PBS) intraperitoneally after suture occlusion. The other groups were administered 25 mg/kg body weight tanshinone IIA (Xi’an Guan Sheng Yuan Co., Ltd., Xi’an, China) by intraperitoneal injection 1, 4, 6 and 12 h, respectively, after the initiation of cerebral ischemia-reperfusion. Tanshinone concentrations of 5–25 mg/kg body weight were found to have a treatment effect in previous studies (14,18). Thus, each rat was given 25 mg/kg body weight tanshinone in the present study.

#### Grouping II and drug administration

A total of >16 male SD rats (Experimental Animal Center for Anhui Medical University, Hefei, China) were randomly divided 6 h and 24 h after the initiation of reperfusion following drug administration. The rats in each group were then further divided into a control subgroup (4 ml PBS) and a tanshinone IIA subgroup (25 mg/kg tanshinone IIA), which were immediately administered their respective treatments following the establishment of the model. The rats were sacrificed 6 h or 24 h after the initiation of reperfusion. This study was carried out in strict accordance with the recommendations in the Guide for the Care and Use of Laboratory Animals of the National Institutes of Health (19). The animal use protocol was reviewed and approved by the Institutional Animal Care and Use Committee (IACUC) of Anhui Medical University.

### Animal model

The arteria carotis communis ligation method adopted by Engel *et al* (20) and Longa *et al* (21) was improved and used in this study. All rats were fasted for 24 h. Then, the rats were anesthetized with 0.4 ml 10% chloral hydrate/100 g body weight by intraperitoneal injection. The rat body temperature was maintained at 36.5–37.5°C (rectal temperature) using an electric blanket during the surgery. The rat skins were generally disinfected and covered with a disinfection cloth. Monofilament nylon sutures measuring 4.0–6.0 cm in length and 0.20–0.22 mm in diameter were used. The head ends of the nylon sutures were touched to the outer flame of an alcohol lamp to control the diameter within the range 0.24 to 0.26 mm. Black labels were used for marking ~2.0 cm from the head end of the nylon sutures. The lines were disinfected with ethanol and then placed in a normal saline solution. A ~20-mm longitudinal incision was made in of the skin of the middle neck. The external carotid artery (ECA), common carotid artery (CCA), internal carotid artery (ICA) and vagus nerve were isolated. The first branch of the ECA was ligated at the distal end. Two wires were crossed under the CCA. One wire was used to ligate the CCA ~5 mm from the bifurcation of the EEA and the ICA. Then, the ICA and the vagus nerve were isolated and the blood flow was blocked using a clip. A thread embolism was inserted into the ICA ~18±2 mm from the incision, which was cut at ~2–4 mm from the bifurcation of the CCA. The clip was then removed. The CCA and the nylon sutures were ligated together, and ~10 mm additional wire was left externally. Tissues were sewn up by layer. The thread embolism was removed 2 h after cerebral ischemia. The temperature of the rats was regulated using filament lamps following the surgery. The rats were placed in a supine position and individually fed following resuscitation. Femoral arterial cannulae for all rats were installed during the surgery to measure the blood pressure and blood pH of the rats.

### Brain blood-flow monitoring

Changes in blood flow in the areas supplied by the right middle cerebral artery were monitored by laser Doppler imaging. Rats were fixed on the operating table in the prone position. The fur was removed from the rat’s head. Then, the head skin was cut open from the bregma up to the skull along the centerline (3 mm back and 5 mm right). The cranium was penetrated using a dental drill. Fiber optic probes were fixed onto the incision of the cranium using biogum. The brain blood flow in the right middle cerebral artery around the ischemic zone was measured prior to and 72 h after the surgery. The reduction in blood flow was calculated. The head incision was sewn up following the measurement.

### Neurobehavioral score

The degree of neurological deficit was estimated by blinded Longa’s method (22). The individuals responsible for recording physical observations and assigning scores did not know the grouping parameters. The scores were assigned as follows: 0, normal condition; 1 point, internal rotation and adduction of the left foreleg; 2 points, resistance reduction of the left side and a tendency for turning to the left; 3 points, imbalanced walking with a tendency of falling to the left; and 4 points, lack of autonomic activities.

### Tetrazolium chloride (TTC) staining

The brains were removed and dyed with TTC (Sigma, St. Louis, MO, USA) 72 h after cerebral ischemia-reperfusion. The percentage of the ischemic tissue weight to the total brain weight represented the degree of cerebral infarction. The brains of rats from each group were removed 24 h after surgery and were placed into a rat brain-slicing mold. Sections 2 mm thick were cut using a surgical knife at ~2 mm from the frontal coronal pole. The sections, which were placed in 0.25% TTC, were incubated in the dark for 30 min at 37°C and then fixed with 4% paraformaldehyde. The white portions observed in the sections were infarcts, whereas the red parts were normal tissues. Photographs of the sections were captured using a digital camera. Two sections of infarct and normal tissues that were isolated based on their colors were weighed. The cerebral infarction volumes were calculated as follows: Cerebral infarction volume (%) = white part weight/total brain weight × 100.

### Western blot analysis

The rats in grouping II were anesthetized with 0.4 ml 10% chloral hydrate 6 h or 24 h after drug administration. The brains were excised and placed on ice. The protein content in the area around the middle cerebral artery infarct was determined using a bicinchoninic acid (BCA) kit (Cwbio Ltd., Beijing, China). Protein lysis buffer was added to the tissue, which was lysed for 30 min at 4°C. Then, the tissue was centrifuged at 13,000 × g for 5 min, and the supernatant was collected. A 15% denaturing polyacrylamide gel was used for gel electrophoresis. The proteins were transferred to a nitrocellulose membrane. The membranes were incubated with rabbit primary antibodies specific for rat Trx-1 (1:1,000; Cell Signaling Technology, Inc., Beverly, MA, USA), goat primary antibodies specific for rat Trx-2 (1:1,000) (R&D Systems, Minneapolis, MN, USA), and β-actin (1:1,000; Cell Signaling Technology, Inc.) overnight at 4°C. Then, the membranes were incubated in anti-rabbit or goat secondary Ig (H+L) antibodies (Promega Corporation, Madison, WI, USA) for 2 h. The proteins were visualized using an electrochemiluminescence (ECL) Western Blotting Detection system (Thermo Scientific Scientific, Inc., Rockford, IL, USA). Images were quantified with Image J Image analysis software (National Institutes of Health, Bethesda, MD, USA).

### Determination of NOS and iNOS activities and NO content

Blood from the rats of Grouping II was drawn through the inner canthus 6 and 24 h after cerebral ischemia-reperfusion. The supernatant was collected into EP tubes following centrifugation and then stored at low temperature. NOS and iNOS activities as well as NO content were measured using a reagent kit (Nanjing Jiancheng Bioengineering Institute, Nanjing, China). The NO concentration was determined using an ultraviolet spectrophotometer at a 540 nm wavelength.

### Statistical analysis

Statistical analysis was performed using SPSS software, version 17.0 (SPSS Inc., Chicago, IL, USA). All data are shown as the mean ± S.D. Mean values of the two groups were compared using an independent sample t-test. Multigroup mean values were compared using repeated analysis of variance (ANOVA). P<0.05 was considered to indicate a statistically significant difference.

## Results

### Neurobehavioral score

**Figure 1:**
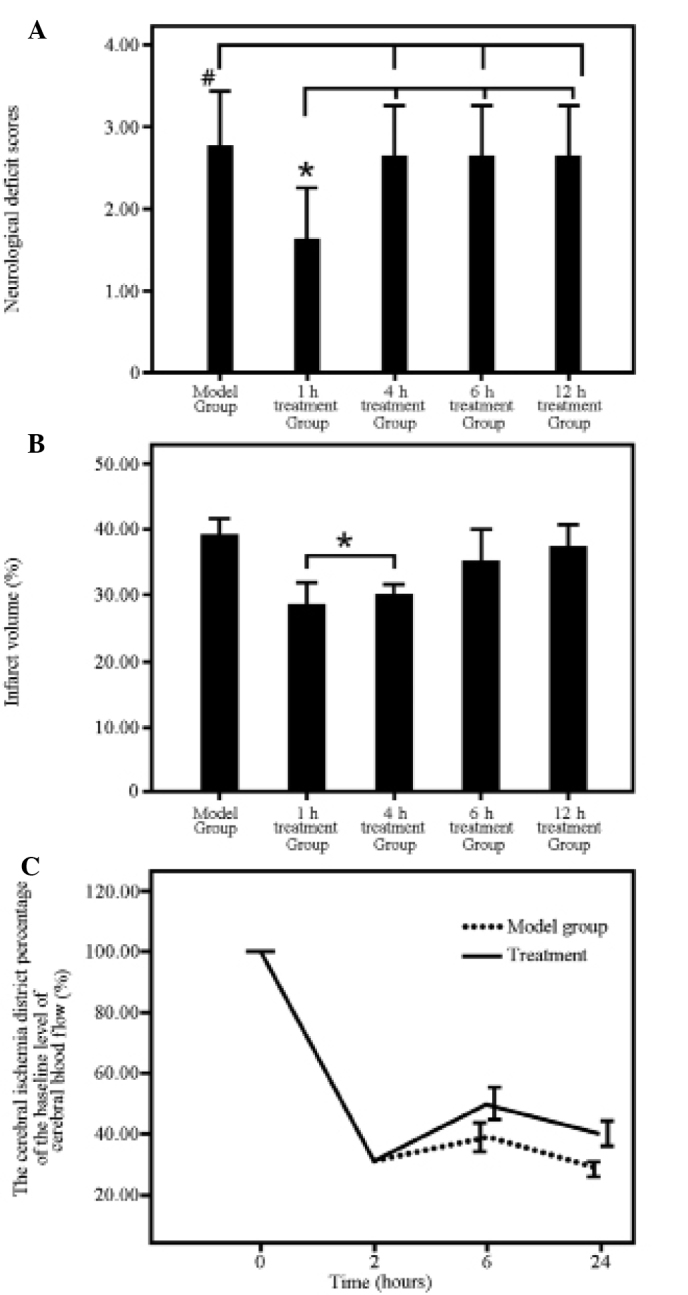
Results of (A) neurobehavioral score, (B) cerebral infarction size and (C) cerebral blood flow change. ^*^The treatment groups with a statistical significance (P<0.05); ^#^The model group with a statistical significance (P<0.05).

The neurobehavior scores of the five groups were obtained 72 h after reperfusion. The neurobehavior scores did not vary significantly with time, which was unexpected. However, statistical significance was observed in the five groups (P=0.037, F=2.88, df=4). A statistically significant difference was observed between the TSA1 group and the following groups: TSA4, TSA6, TSA12 and control (P<0.05). A statistically significant difference was also observed between the control group and the following groups: TSA4, TSA6, TSA12 and TSA1 (P<0.05). No significant differences were found among the TSA4, TSA6 and TSA12 groups (Fig. 1A).

These results indicate that when there was a delay time of >4 h in the administration of tanshinone IIA to the model rats, tanshinone IIA was not able to reduce the neurobehavior score.

### Cerebral infarction volume

The cerebral infarction volume results are shown in Fig. 1B. The rats were sacrificed 72 h after the initiation of reperfusion. The cerebral infarction volumes were statistically significant in the five groups (F=11.036 and P<0.001). The cerebral infarction volumes of the TSA1 and TSA4 groups were significantly lower than those of the control, TSA6 and TSA12 groups (P<0.05). No significant differences were observed among the control, TSA6 and TSA12 groups (P>0.05).

### Trx-1 and Trx-2 expression

**Figure 2:**
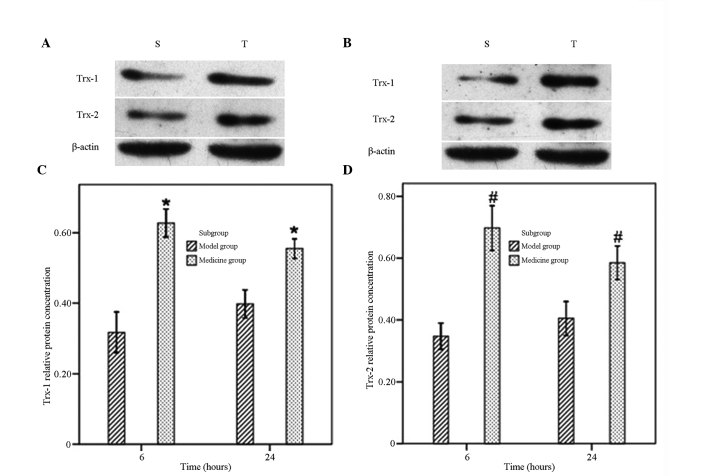
Protein expression levels of Trx-1 and Trx-2. Trx, thioredoxin. ^*^Treatment groups with statistical significance in Trx-1 (P<0.05); ^#^Treatment group with a statistical significance in Trx-2 (P<0.05). S, control subgroup; T, tanshinone IIA subgroup.

The expression levels of Trx-1 and Trx-2 of the rats in the tanshinone IIA subgroups of the 6 and 24 h reperfusion groups were significantly increased compared with those of the control group (P<0.05; Fig. 2).

### NOS and iNOS activities and NO content

Compared with those of the respective model control subgroup, the NOS activity and the NO content of the tanshinone IIA subgroups of the 6 h and 24 h reperfusion groups were significantly decreased (P<0.05). The iNOS activity of the tanshinone IIA subgroup of the 24 h reperfusion group was also significantly decreased compared with that of the model control (P<0.05; Table I).

### Cerebral blood flow changes

The cerebral blood flow was not statistically significantly different between the control subgroup and the tanshinone IIA subgroup in the 2 h reperfusion group: 31.62±1.48 and 31.00±1.53%, respectively. The cerebral blood flow was significantly different between the control subgroup and tanshinone IIA subgroup in the 6 h reperfusion group: 39.10±2.82 and 49.97±3.41%, respectively. The cerebral blood flow was also significantly different between the control subgroup and the tanshinone IIA subgroup in the 24 h reperfusion group: 28.51±1.42 and 40.20±2.42%, respectively (Fig. 1C). The Doppler results indicate that tanshinone IIA possesses a vessel expansion effect.

## Discussion

The cerebral ischemia-reperfusion model is a recognized cerebral ischemic model (22). The protective mechanism and treatment time window of tanshinone IIA in the cerebral ischemia-reperfusion model were investigated in the present study. A better protective effect was observed when tanshinone IIA was administered early. Moreover, different protective effects were observed when tanshinone IIA was administered at different treatment time windows of the cerebral ischemic disease. This protective effect may be associated with improvements in cerebral blood flow, anti-radical and anti-inflammatory activities. Previous studies indicated that tanshinone IIA (20 mg/kg body weight) had a protective effect on a cerebral ischemia-reperfusion model, whereby the neurobehavior score was significantly reduced, encephaledema was relieved and the cerebral infarction volume was decreased (17,23). Tang *et al* (24) found that tanshinone IIA significantly relieved encephaledema and cerebral infarction in a rat model at 24 h reperfusion. A dose of ~30 mg/kg body weight tanshinone IIA showed a marked treatment effect. Thus, the current study used a 25 mg/kg body weight dose of tanshinone IIA to investigate the treatment effect and treatment mechanism of tanshinone IIA.

The results indicate that tanshinone IIA reduced the neurobehavior score, decreased the cerebral infarction volume and improved the cerebral blood flow. Different protective effects were observed when tanshinone IIA was administered at various treatment time windows. Moreover, the dose of 25 mg/kg body weight tanshinone IIA also conferred a great protective effect on the brain. Tanshinone IIA treated brain reperfusion injury within a certain time window, which affected the prognosis. Tanshinone IIA significantly relieved cranial nerve function injury within 1 h of reperfusion (P<0.05 for the TSA1 group compared with the other groups). This result demonstrates that tanshinone IIA is able to relieve cranial nerve function injury. However, the cerebral infarction volumes in the 1 h and 4 h reperfusion groups were significantly lower than those in the other groups (P<0.05), which indicates that different treatment time windows may affect the cerebral infarction volume. However, this result was inconsistent with the neurobehavior score results in which statistical significance was only observed between the TSA1 group and the other groups. The possible reasons are as follows. First, the two methods were inconsistent. Secondly, the neurobehavior scoring method used may not have been accurate. These results suggested different issues to the authors concerning the treatment of the cerebral ischemia-reperfusion model. It was unclear whether tanshinone IIA had an effect on patients whose disease course exceeded the treatment time window. The treatment effect of tanshinone IIA on rats 4 h after reperfusion was not found to be ideal in this study. However, the authors considered that tanshinone IIA was able to improve blood flow and certain other indices that are important for patients. Thus, a comprehensive clinical study should be performed to obtain a conclusion concerning the effectiveness of tanshinone II as a treatment for cerebral apoplexy.

The rescue of nerve cells in the ischemic penumbra is a main approach for the treatment of cerebral infarction. Apoptosis plays an important role in nerve cell injury following reperfusion (26–28). Previous studies have confirmed that tanshinone IIA is able to inhibit the apoptosis of nerve cells (29,30). However, the mechanism by which it inhibits apoptosis is not yet understood. In further studying the protective effect of tanshinone IIA on the brain, the effect of tanshinone IIA on Trx and NO were also investigated in the present study. NO is an important messenger and effector molecule that possesses neurotransmitter and neuromodulator functions and participates in physiological and pathological activities. NO may have a damaging or protective effect depending on ischemia time, reperfusion time and the type of cell producing NO. NOS, a key enzyme synthesizing NO, exists in three types: nNOS, eNOS, and iNOS. nNOS and eNOS is activated by calcium ions and calmodulin, whereas iNOS is activated by inflammatory factors and endotoxin. The action time of iNOS is prolonged, and iNOS is able to catalyze the synthesis of a large amount of NO (7). Numerous studies have reported that NOS is the key deciding factor in the dual effect of NO during cerebral ischemia-reperfusion injury (Liu, Dohare, Blomgren). The activity of NOS increases at all stages of cerebral ischemia. The different NOS types have different functions. eNOS activity increases and catalyzes NO synthesis, thereby expanding the blood vessels of the brain and increasing brain blood flow to protect the brain at the very early stage of cerebral infarction (<2 h). The protective effect disappears 2 h after cerebral infarction. nNOS is able to catalyze the synthesis of a large amount of NO, which has a toxic effect on the brain in the early period of cerebral infarction (2–6 h). iNOS is also able to catalyze the synthesis of a large amount of NO, thus increasing the toxicity of glutamic acid and leading to delayed neuronal damage in the later period of cerebral infarction (>6 h) (8).

Compared with those of the control subgroup, the NOS activity and the NO levels in the tanshinone IIA subgroups of the 6 and 24 h reperfusion groups were significantly decreased (P<0.05). The iNOS activity in the tanshinone IIA subgroup of the 24 h reperfusion group was significantly decreased (P<0.05; Table I). These results indicate that tanshinone IIA significantly decreased nNOS activity in the early period of cerebral infarction. However, the changes in iNOS activity were not statistically significant in the early period. The possible reason for this is that nNOS plays an important role in early cerebral infarction. By contrast, the activity of iNOS did not markedly increase. Thus, the effect of tanshinone IIA on iNOS was not clear. However, tanshinone IIA had a significant effect on iNOS in the later period of cerebral infarction. Moreover, nNOS activity, iNOS activity and NO content significantly increased with time extension, which indicates that increased NO levels may be achieved as a result of the increases in nNOS and iNOS activities. NO combines with N-methyl D-aspartate receptors, which leads to the opening of calcium channels and increased intracytoplasmic calcium content (Han). Cells eventually become overloaded with intracellular calcium and a cascade of injuries is induced. iNOS levels increase with reperfusion injury development, inflammatory cell infiltration, and increases in the levels of TNF-α and IL-β, which mediates the neurovirulence effect and causes further injury to the neurons (30).

The free-radical theory is of concern in the pathogenesis of central nervous system diseases. Once free radicals accumulate and are not eliminated in a timely manner, the cell structure may be damaged. As a result, cell function may be lost, leading to central nervous system diseases. Trx is a type of a redox active micromolecular protein that has many important biological functions, such as mediating the transport of H^+^ ions in systems involving NADPH and thioredoxin reductase, playing a role in oxidoreduction as protein disulfide bond, acting as a biomarker of oxidative stress, and participating in oxidative stress and cell apoptosis. Trx includes Trx-1 and Trx-2 subtypes. A system composed of Trx-1, Trx-2 and NADPH in histiocytes eliminates free radicals and protects brain tissue (4). The mRNA expression levels of Trx-1 and Trx-2 around the ischemia area are significantly increased in the brain transient ischemia model created by the blockage of the middle cerebral artery. Trx not only eliminates free radicals but also downregulates caspase-3 expression, which inhibits apoptosis (2,10,11,31). Moreover, certain studies have found that Trx-2 when expressed at high levels more strongly resists the toxicity induced by mitochondria complex III (11,32).

In the present study, the Trx-1 and Trx-2 expression levels in the tanshinone IIA subgroups (6 and 24 h cerebral ischemia-reperfusion) were significantly increased compared with those in the respective control subgroups (P<0.05), indicating that tanshinone IIA increased the protein expression levels of Trx-1 and Trx-2. Based on the Trx expression results of the 6 h reperfusion group, tanshinone IIA use at an early stage may enhance the capacity for free-radical elimination. Moreover, if Trx is expressed earlier, the molecules that eliminate free radicals are likely to be better activated. The Trx induced by tanshinone IIA exerts a protective effect on nerve cells through free-radical resistance. Cerebral infarction volume results in the present study also indicated that tanshinone IIA possesses an anti-apoptotic effect. Since improvement of the microcirculation of the ischemic region is an approach for the treatment of cerebral infarction, the present study also investigated whether tanshinone IIA was able to improve the microcirculation. The following findings were established. First, the cerebral blood flow was not significantly different between the control and tanshinone IIA subgroups in the 2 h reperfusion group, at 31.62±1.48 and 31.00±1.53%, respectively. Secondly, the cerebral blood flow in the control and tanshinone IIA subgroups of the 6 h reperfusion group were significantly different at 39.10±2.82 and 49.97±3.41%, respectively. Thirdly, the cerebral blood flow in the control and tanshinone IIA subgroups in the 24 h reperfusion group were significantly different at 28.51±1.42 and 40.20±2.42%, respectively. These results indicate that tanshinone IIA is able to induce the expansion of blood vessels.

In conclusion, tanshinone IIA has a protective effect on cranial nerves when administered at the early stage of cerebral ischemia. The protective effect is associated with improvements in cerebral blood flow as well as anti-oxygen radical and anti-inflammatory activities.

## Figures and Tables

**Table I:** NO and the activity of its induced enzyme.

Time (h)	Analyte	Model group	Drug group
6	NOS (U/g)	19.93±2.21	16.30±1.68^a^
6	iNOS (U/g)	5.40±0.83	4.67±0.55
6	NO (μmol/g)	25.23±2.13	20.81±2.01^a^
24	NOS (U/g)	22.16±2.46	14.91±1.64^a^
24	iNOS (U/g)	6.92±0.56	5.43±0.96^a^
24	NO (μmol/g)	30.74±2.14	22.15±2.22^a^

a P<0.05 versus the model group.

## References

[1] Chollet F, Cramer SC, Stinear C (2013). Pharmacological therapies in post stroke recovery: recommendations for future clinical trials. J Neurol.

[2] Candelario-Jalil E (2009). Injury and repair mechanisms in ischemic stroke: considerations for the development of novel neurotherapeutics. Curr Opin Investig Drugs.

[3] Lo EH, Dalkara T, Moskowitz MA (2003). Mechanisms, challenges and opportunities in stroke. Nat Rev Neurosci.

[4] Mustacich D, Powis G (2000). Thioredoxin reductase. Biochem J.

[5] Kishimoto K, Li RC, Zhang J (2010). Cytosolic phospholipase A2 alpha amplifies early cyclooxygenase-2 expression, oxidative stress and MAP kinase phosphorylation after cerebral ischemia in mice. J Neuroinflammation.

[6] Stoyanova II, Lazarov NE (2005). Localization of nitric oxide synthase in rat trigeminal primary afferent neurons using NADPH-diaphorase histochemistry. J Mol Histol.

[7] Bredt DS, Snyder SH (1989). Nitric oxide mediates glutamate-linked enhancement of cGMP levels in the cerebellum. Proc Natl Acad Sci USA.

[8] Kawase M, Kinouchi H, Kato I (1996). Inducible nitric oxide synthase following hypoxia in rat cultured glial cells. Brain Res.

[9] Hirota K, Nakamura H, Masutani H, Yodoi J (2002). Thioredoxin superfamily and thioredoxin-inducing agents. Ann N Y Acad Sci.

[10] Berk BC (2007). Novel approaches to treat oxidative stress and cardiovascular diseases. Trans Am Clin Climatol Assoc.

[11] Billiet L, Furman C, Cuaz-Pérolin C (2008). Thioredoxin-1 and its natural inhibitor, vitamin D3 up-regulated protein 1, are differentially regulated by PPARalpha in human macrophages. J Mol Biol.

[12] Watson HW, Yang X, Choi YE, Jones DP, Kehrer JP (2004). Thioredoxin and its role in toxicology. Toxicol Sci.

[13] Xu W, Yang J, Wu LM (2009). Cardioprotective effects of tanshinone IIA on myocardial ischemia injury in rats. Pharmazie.

[14] Lam BY, Lo AC, Sun X, Luo HW, Chung SK, Sucher NJ (2003). Neuroprotective effects of tanshinones in transient focal cerebral ischemia in mice. Phytomedicine.

[15] Dong K, Xu W, Yang J, Qiao H, Wu L (2009). Neuroprotective effects of Tanshinone IIA on permanent focal cerebral ischemia in mice. Phytother Res.

[16] Adams JD, Wang R, Yang J, Lien EJ (2006). Preclinical and clinical examinations of *Salvia miltiorrhiza* and its tanshinones in ischemic conditions. Chin Med.

[17] Liu LL, Zhang XL, Wang L (2010). The neuroprotective effects of Tanshinone IIA are associated with induced nuclear translocation of TORC1 and upregulated expression of TORC1, pCREB and BDNF in the acute stage of ischemic stroke. Brain Res Bull.

[18] Chen X, Zhou ZW, Xue CC, Li XX, Zhou SF (2007). Role of P-glycoprotein in restricting the brain penetration of tanshinone IIA, a major active constituent from the root of *Salvia miltiorrhiza* Bunge, across the blood-brain barrier. Xenobiotica.

[19] National Research Council (2001). Guide for the care and use of laboratory animals.

[20] Engel O, Kolodziej S, Dirnagl U, Prinz V (2011). Modeling stroke in mice - middle cerebral artery occlusion with the filament model. J Vis Exp.

[21] Longa EZ, Weinstein PR, Carlson S, Cummins R (1989). Reversible middle cerebral artery occlusion without craniectomy in rats. Stroke.

[22] Hoffman GE, Merchenthaler I, Zup SL (2006). Neuroprotection by ovarian hormones in animal models of neurological disease. Endocrine.

[23] Wang LN, Zhang XJ, Liu LL (2010). Tanshinone IIA down-regulates HMGB1, RAGE, TLR4, NF-kappaB expression, ameliorates BBB permeability and endothelial cell function, and protects rat brains against focal ischemia. Brain Res.

[24] Tang C, Xue HL, Bai CL, Fu R, Wu AH (2010). The effects of Tanshinone IIA on blood-brain barrier and brain edema after transient middle cerebral artery occlusion in rats. Phytomedicine.

[25] Chen D, Tang J, Khatibi NH (2011). Treatment with Z-ligustilide, a component of *Angelica sinensis*, reduces brain injury after a subarachnoid hemorrhage in rats. J Pharmacol Exp Ther.

[26] Cavallucci V, D’Amelio M (2011). Matter of life and death: the pharmacological approaches targeting apoposis in brain diseases. Curr Pharm Des.

[27] Nizamutdinova IT, Jin YC, Kim JS (2008). Paeonol and paeoniflorin, the main active principles of *Paeonia albiflora*, protect the heart from myocardial ischemia/reperfusion injury in rats. Planta Med.

[28] Liu Y, Wang L, Li X, Lv C, Feng D, Luo Z (2010). Tanshinone IIA improves impaired nerve functions in experimental diabetic rats. Biochem Biophys Res Commun.

[29] Zhang WJ, Feng J, Zhou R (2010). Tanshinone IIA protects the human blood-brain barrier model from leukocyte-associated hypoxia-reoxygenation injury. Eur J Pharmacol.

[30] Anctil M, Poulain I, Pelletier C (2005). Nitric oxide modulates peristaltic muscle activity associated with fluid circulation in the sea pansy *Renilla koellikeri*. J Exp Biol.

[31] Das KC (2004). Thioredoxin system in premature and newborn biology. Antioxid Redox Signal.

[32] Nalvarte I, Damdimopoulos AE, Spyrou G (2004). Human mitochondrial thioredoxin reductase reduces cytochrome *c* and confers resistance to complex III inhibition. Free Rad Biol Med.

[33] Liu R1, Gao M, Yang ZH (2008). Pinocembrin protects rat brain against oxidation and apoptosis induced by ischemia-reperfusion both in vivo and in vitro. Brain Res.

[34] Dohare P, Varma S, Ray M (2008). Curcuma oil modulates the nitric oxide system response to cerebral ischemia/reperfusion injury. Nitric Oxide.

[35] Blomgren K, Hagberg H (2006). Free radicals, mitochondria, and hypoxia-ischemia in the developing brain. Free Radic Biol Med.

[36] Han JD (2012). The investigation of nerveprotective effect and mechanism of TongLuoHuaTan capsule and its active ingredient on cerebral ischemia reperfusion injury. Doctoral dissertation.

